# Supplementation with alpha-glycerol monolaurate during late gestation and lactation enhances sow performance, ameliorates milk composition, and improves growth of suckling piglets

**DOI:** 10.1186/s40104-023-00848-x

**Published:** 2023-04-05

**Authors:** Longxian Li, Huakai Wang, Shuang Dong, Yongxi Ma

**Affiliations:** State Key Laboratory of Animal Nutrition, College of Animal Science and Technology, China Agricultural University, Beijing, China

**Keywords:** Alpha-glycerol monolaurate, Fatty acid, Immune status, Microbiota, Sows, Suckling piglets

## Abstract

**Background:**

Physiological changes during lactation cause oxidative stress in sows, reduce immunity, and hamper the growth capacity of piglets. Alpha-glycerol monolaurate (α-GML) has potential for enhancing the antimicrobial activity of sows and the growth of suckling piglets.

**Methods:**

Eighty sows were allocated randomly to four groups: basal diet and basal diets supplemented with 500, 1000, or 2000 mg/kg α-GML. The experiment started on d 85 of gestation and lasted until piglets were weaned on d 21 of lactation. The number of live-born piglets was standardized to 12 ± 1 per sow on day of parturition. On d 0 and 21 of lactation, body weight of piglets was measured and milk samples were obtained from sows, and serum samples and feces from piglets were obtained on d 21.

**Results:**

Feed intake, backfat loss, and weaning estrus interval did not differ among the four groups of sows. Maternal α-GML supplementation increased (*P* < 0.05) the body weight of piglets at weaning and the apparent total tract digestibility of crude fat of sows. The immunoglobulin A and immunoglobulin G levels were greater (*P* < 0.05) in a quadratic manner in the milk of sows as dietary α-GML increased. Concerning fatty acid profile, C12:0, C15:0, C17:0, C18:2n6c, C18:3n3, C24:0, and C22:6n3 were higher (*P* < 0.05) in linear and quadratic manners in colostrum of sows-fed α-GML diets compared with the control sows. There was lower (*P* < 0.05) n-6:n-3 polyunsaturated fatty acid ratio in milk than in the control sows. Maternal α-GML increased the abundance of Firmicutes (*P* < 0.05) and decreased the abundance of Proteobacteria (*P* < 0.05) of piglet fecal microbiota.

**Conclusions:**

Dietary supplementation with α-GML improved milk immunoglobulins and altered fatty acids of sows, thereby improving the health of piglets.

## Background

Litter size and weaning weights are the main parameters that define productivity and profitability on pig barn [1]. Improvements in swine genetics [2], management [3], and nutrition [4] have increased the number of weaned piglets per litter [5], which are associated with larger litter size, pre-weaning survival and weaning weight. Three-quarters of fetal weight gain occurs in the last quarter of pregnancy, during which sufficient nutrient is required to meet the needs of the sow [6]. It is important to provide adequate nutrients or nutritional interventions through late pregnancy and lactation.

Studies have found that dietary supplementation of fatty acids during late pregnancy and/or lactation can increase the overall litter growth and pig weaning weights [7], reduce body weight loss during lactation and shorten the weaning-to-estrus interval [6]. Furthermore, the type and amount of fat in daily feed rations also affect the sow milk yield and the milk fatty acids profile [8]. Alpha-glycerol monolaurate (α-GML) is an α-monoglyceride of medium-chain fatty acid (MCFA) lauric acid and is recognized by the US Food and Drug Administration (FDA) as a Generally Recognized as Safe (GRAS) food emulsifier [9]. As a natural and edible lipid, α-GML possesses multiple pharmacological and biological activities, including excellent antibacterial and antiviral properties. The strong antibacterial properties of GML make it widely used in food preservation and medicine [10–12]. Moreover, in vitro study showed that GML inhibited the infectivity and transmission of the African swine fever virus (ASFv) in feed [13]. A recent study reveals that GML can be used as an effective feed additive to improve animal growth performance and quality control [14]. Our previous study has demonstrated that dietary 1000 mg/kg α-GML supplementation significantly reduces diarrhea rate, improves intestinal morphology, nutrient digestibility, antioxidant capacity, and immune status, and ameliorates gut microbiota in weaned piglets [15]. It is reported that the addition of 2000 mg/kg GML in a low protein diet could improve the intestinal tight junction barrier function and immune status of weaned piglets [16]. However, whether α-GML can improve the health of suckling piglets through the mother is not yet conclusive. Therefore, the present study aimed to determine the effect of maternal α-GML supplementation during gestation and lactation on the reproductive performance of sows, the fatty acids profile of milk, the growth performance, the serum immunoglobulins, and the fecal microbiota of piglets.

## Methods

The experimental protocol of this study was following the Guide for the Care and Use of Laboratory Animals prepared by the Institutional Animal Care and Use Committee of China Agricultural University. This experiment was conducted in the FengNing Swine Research Unit of China Agricultural University (Academician Workstation in Chengde Jiuyun Agricultural and Livestock Co., Ltd., Hebei, China). The product α-GML was provided by Zhejiang Libiduo Biotechnology Co., Ltd., and the α-GML accounted for 85% of the products.

### Animals and experimental design

Fifth or sixth parity sows (*n* = 80; Landrace × Large White) with similar backfat (BF) thickness were assigned randomly to one of four treatments: control diet (corn-soybean basal diet, *n* = 20), 500 mg/kg α-GML diet (basal diet + 500 mg/kg α-GML, *n* = 20), 1000 mg/kg α-GML diet (basal diet + 1000 mg/kg α-GML, *n* = 20), and 2000 mg/kg α-GML diet (basal diet + 2000 mg/kg α-GML, *n* = 20). Each treatment (Table 1) consisted of a gestation and a lactation diet and were formulated to meet nutritional requirements of late gestation (d 85 to 107 of gestation) and lactation (d 107 of gestation to weaning) sows according to the recommendations by the National Research Council [17]. Sows were housed individually in stalls from d 85 to 107 of gestation on partially slatted concrete floors and ingested a total of 2.5 kg diet daily (9:00 and 15:00). Then, sows were moved to farrowing stalls on d 107 of gestation and ingested 2.76 kg of daily diet (9:00 and 15:00) from d 107 of gestation to parturition. Sows were offered approximately 0.5 kg on the first day after farrowing and then fed daily feed allotment was increased 1.0 kg more every day until maximum feed intake was reached. When maximum feed intake was achieved, sows were allowed ad libitum access to feed. Throughout the experiment, sows and piglets were allowed ad libitum access to water. On the day of farrowing (day = 0), litter size was adjusted to 12 ± 1 piglets by cross-fostering within dietary treatment. The ambient temperature in the farrowing room for sows was maintained at 20–23 ℃. Heat lamps were used to provided heat to neonatal pigs. The suckling piglets underwent routine tooth clipping, tail removal, subcutaneous iron dextran injections, and immunization. During the entire experiment, sow's milk was the sole food source for suckling piglets. Feed intake of the sows was recorded daily to allow calculation of average daily feed intake (ADFI) during the entire lactation period, and piglets were weighed at birth and d 21 of lactation. The growth performance and survival rates of sucking piglets were determined. Backfat thickness (P2, 6 cm from the midline at the head of the last rib) of sows at d 110 of gestation and d 21 of lactation was measured using an ultrasonic device (Piglog105; SFK Technology A/S, Helver, Denmark) to assess the body condition of sows.

**Table 1:** Ingredient composition and calculated nutrient concentrations of gestation and lactation basal diets (as-fed basis)

Item	Late gestation	Lactation
Ingredient composition, %		
Corn	57.50	61.50
Soybean meal	15.50	20.50
Wheat bran	20.00	10.00
Fish meal	2.00	2.00
Soybean oil	2.00	3.00
Limestone	1.00	1.00
Dicalcium phosphate	1.05	1.00
Salt	0.30	0.30
*L*-Lysine-HCl,78%	-	0.05
Choline chloride	0.15	0.15
Premix^1^	0.50	0.50
Total	100.00	100.00
Nutrient concentrations^2^		
Digestible energy, MJ/kg	14.61	15.09
Crude protein, %	16.21	17.01
SID Lysine, %	0.85	0.98
Calcium, %	0.93	0.93
Total phosphorus, %	0.73	0.67
Available phosphorus, %	0.52	0.50

^1^The premix provided the following per kg of the diet: Vitamin A, 25,000 IU; Vitamin D_3_, 5000 IU; Vitamin E, 12.5 IU; Vitamin K_3_, 2.5 mg; Thiamin, 8.0 mg; Pyridoxine, 3.0 mg; Vitamin B_12_, 15 μg; Riboflavin, 6.0 mg; Niacin, 17.5 mg; *D*-Pantothenic acid, 12.5 mg; Folic acid, 0.25 mg; Biotin, 0.1 mg; Choline chloride, 0.4 mg; Fe, 165 mg; Cu, 16.0 mg; Zn, 165 mg; Mn, 30 mg; I, 0.3 mg; Se, 0.3 mg

^2^Calculated values

### Collection of feed and Feces from sows

Approximately 1 kg of gestation and lactation feed were collected weekly during the experiment. From d 19 to 21 of lactation, six sows each group were sued to collect fresh feces by the rectal palpation method. Samples were pooled within sows over the three collection days and immediately frozen at −20 °C. Feces were dried at 65 °C for 72 h. All feed and feces samples were ground to pass through a 1-mm sieve and stored at −20 °C for further analysis.

### Collection of colostrum and milk from sows

Six sows from each group were randomly selected by injection of 10 IU oxytocin via the ear vein to induce milk letdown into the teat canal on d 0 (colostrum, within 2 h after starting delivery of the sows) and 21 (milk) of lactation. Milk samples were collected from different mammary glands (front, middle, and rear), and equal volumes (15 mL) of milk from three glands of a sow were mixed, and the samples were stored at –20 ℃ for subsequent analysis.

### Collection of blood samples from piglets

Blood samples (2 mL) were obtained from six randomly piglets each group via the jugular vein using non-heparinized vacutainer tubes on d 21 of lactation. Serum samples were harvested for each blood sample after centrifugation at 3000 × *g* for 15 min, and were stored at –20 ℃ frige for subsequent analysis.

### Collection of fecal samples from piglets

Six piglets each group were used to collect fresh feces by the rectal palpation method on d 21 of lactation. Fecal samples were immediately frozen in liquid nitrogen and placed at −80 ℃ frige for subsequent analysis.

### Analysis of nutrient digestibility

Gross composition of diets and feces were determined according to the Association of Official Analytical Chemists methods [18]. Moisture, ash, crude protein (CP), and crude fat (CF) of feed and fecal samples were analyzed using AOAC methods 930.15, 942.05, 990.03, and 996.01, respectively. Gross energy (GE) was determined using an adiabatic bomb calorimeter (Parr 1281, Automatic Energy Analyzer; Moline, IL, USA). Apparent total tract digestibility (ATTD) of dry matter (DM), CP, CF, and GE was determined by the indigestible marker method using inherent acid insoluble ash (AIA) as the indigestible marker. The AIA concentrations in diets and feces were determined based on procedures described by Prawirodigdo et al. [19]. The ATTD of nutrients was calculated using the following equation:

$$\textit{ATTD of nutrients}={\textit{1-(AIA}}_\textit{diet}\times{\textit{Nutrients}}_\textit{feces}\textit{)}/{\textit{(AIA}}_\textit{feces}\times{\textit{Nutrients}}_\textit{diet}\textit{)}$$

### Analysis of immunoglobulin in colostrum and milk

The concentrations of immunoglobulin A (IgA), immunoglobulin G (IgG), and immunoglobulin M (IgM) in colostrum and milk were determined according to Che et al. [20]. Briefly, the optical density values of IgA, IgG and IgM standards were measured at 340, 700, and 340 nm using a UV–VIS recording spectrophotometer (UV-2401PC, Shimadzu Co., Ltd., Kyoto, Japan), and the concentration of each immunoglobulin was calculated by standard curve.

### Analysis of fatty acid profiles in colostrum and milk

For analysis of fatty acid composition, milk samples (1 g) were transferred to a 25-mL Teflon-lined tube and neutralized with 4 mL of n-Hexane:isopropanol (3:2) and 2 mL of sodium sulfate (6.67%). After centrifugation at 5000 × *g* for 10 min, all supernatants were transferred to a 20-mL hydrolysis tube, 200 µL of C11:0 internal standard was added, and dry with mixed nitrogen. Add 4 mL of hydrochloric acid methanol (3 mol/L) solution to the hydrolysis tube and tighten the cap, then reflux in a water bath at 80 ℃ for 2 h. After the water bath, 5 mL of 7% K_2_CO_3_ and 3 mL of hexane were added to the hydrolytic tube, mix by vortexing, centrifuged at 1000 × *g* for 1 min, and about 1 mL of the upper liquid was filtered into a 1.5-mL glass bottle with a filter (filter pore size of 0.22 μm). Finally, the fatty acid methyl ester dissolved in the supernatant was analyzed by gas chromatography–mass spectrometry using Agilent 7890 B (Agilent Technologies, Palo Alto, CA, USA) gas chromatograph and Agilent J&W DB-23 column (60 m × 250 μm ID, 0.25 μm) column. Fatty acids were expressed as the proportion of each individual fatty acid to the total amount of all fatty acids in the sample. n-3 polyunsaturated fatty acid (PUFA), n-6 PUFA, and n-6:n-3 PUFA ratio were calculated.

### Analysis of immunoglobulin in serum

Concentrations of IgA, IgG, and IgM were analyzed using ELISA kits validated for swine (Nanjing Jiancheng Biology Co., Ltd., Nanjing, China) according to methods described [6].

### Fecal microbial flora composition

Total feces bacterial DNA (*n* = 6) was extracted according to the manufacturer's instructions of the QIAamp DNA stool extraction kit (Qiagen, Hilden, Germany). The V3-V4 hypervariable regions of bacterial 16S rRNA were amplified by a PCR system using universal primers 338F (5'-ACTCCTACGGGAGGCAGCAG-3') and 806R (5'-GGACTACHVGGGTWTCTAAT-3'). The PCR amplification procedures were set in ABI GeneAmp® 9700 system (Applied Biosystems, Foster City, CA, USA) as follows: predenaturation (95 ℃, 3 min), amplification (95 ℃, 30 s; 55 ℃, 30 s; 72 ℃, 45 s; a total of 27 cycles), extended (72 ℃, 10 min) [21]. The paired-end reads of pooled purified amplicons were sequenced on the Illumina MiSeq PE300 platform (Illumina, San Diego, CA, USA). The original sequences were demultiplexed, quality filtered, trimmed, and denoised using Trimmomatic and merged according to the overlapping relationship by FLASH software (v1.2.11, http://ccb.jhu.edu/software/FLASH/index.shtml) [22]. The operational taxonomic units (OTUs) with 97% similarity threshold were clustered using UPARSE (version 7.1, http://drive5.com/uparse/) and their representative sequence categorized and analyzed by the RDP Classifier (http://rdp.cme.msu.edu/) against the Silva (SSU128) 16S rRNA database with a confidence threshold of 70% [17]. The composition and structure of fecal microbiota were analyzed according to the standardized OUT with the QIIME software (version 1.8.0) [23]. The linear discriminant analysis (LDA) effect size (LEfSe) algorithm was applied to identify specific taxa from phylum to genus level among each group of samples.

### Statistical analysis

All data were analyzed using the one-way ANOVA procedure of IBM SPSS statistical software (version 26.0, IBM Crop, Armonk, NY, USA). Each sow (litter) or piglet was considered as the experimental unit. Data were evaluated for normality and homoscedasticity by the Shapiro–Wilk and Levene's tests, respectively. The linear and quadratic effects of different α-GML levels were determined using orthogonal polynomials for reproductive performance, concentrations of immunoglobulin and fatty acid composition of sows in milk, and serum antioxidant capacity and immunoglobulin levels of piglets. The bacterial community at the phylum, genus, and species level were analyzed by non-parametric factorial Kruskal–Wallis test and unpaired Wilcoxon Comparison test. *P* < 0.05 was considered significant, and 0.05 ≤ *P* < 0.10 was classified as trends.

## Results

### Reproductive performance

There were no differences (*P* > 0.05) in ADFI of sows and weaning estrus interval among the four groups (Table 2). Litter size at weaning and average weight at birth of piglets did not differ (*P* > 0.05) among treatments. Litter weight at birth and weaning, and average weight at weaning of piglets increased in a quadratic manner (*P* < 0.05) as dietary α-GML content increased.

**Table 2:** Effects of α-glycerol monolaurate (α-GML) supplementation on reproductive performance of sows

Item	Dietary α-GML level, mg/kg				SEM	*P*-value		
	0	500	1000	2000		Treatment	Linear	Quadratic
Sows								
ADFI, kg/d	6.12	6.21	6.38	6.36	0.05	0.20	0.07	0.33
Backfat thickness on d 110 of gestation	19.67	19.38	19.81	19.94	0.56	0.98	0.98	0.94
Backfat loss, mm	1.82	1.67	1.76	1.39	0.23	0.92	0.54	0.86
Weaning estrus interval, d	4.65	4.95	5.05	5.17	0.12	0.47	0.15	0.29
Litter size (*n*)								
At birth	13.24	14.41	14.21	13.43	0.30	0.40	0.21	0.44
At weaning	11.60	11.95	11.86	11.71	0.11	0.66	0.95	0.27
Litter weight, kg								
At birth	13.81	16.99	16.25	16.51	0.41	< 0.05	0.08	< 0.05
At weaning	68.23	75.08	75.97	72.95	1.14	0.06	0.28	< 0.05
Piglets weight, kg								
At birth, kg	1.27	1.33	1.29	1.27	0.02	0.57	0.68	0.38
At weaning, kg	5.89	6.23	6.40	6.20	0.08	0.13	0.21	< 0.05

Values are means with SEM, *n* = 20 sows/treatment group

*ADFI* Average daily feed intake

### Nutrient digestibility

Dietary supplementation with α-GML increased (*P* < 0.05) ATTD of CF compared to the control group (Table 3).

**Table 3:** Effects of α-glycerol monolaurate (α-GML) supplementation during late gestation and lactation on apparent total tract digestibility of nutrients (%) of sows

Item	Dietary α-GML level, mg/kg				SEM	*P*-value		
	0	500	1000	2000		Treatment	Linear	Quadratic
Dry matter	86.80	87.59	88.25	86.91	0.23	0.08	0.69	0.08
Crude protein	88.21	88.73	89.58	88.33	0.22	0.11	0.87	0.07
Crude fat	67.69	69.91	73.22	69.72	0.73	0.05	0.96	0.34
Gross energy	88.34	88.64	89.37	88.65	0.20	0.17	0.90	0.13

Values are means with SEM, *n* = 6 sows/treatment group

### Concentrations of immunoglobulin of sows in colostrum and milk

The IgA and IgG levels in the colostrum and IgA levels in the milk increased in a quadratic manner (*P* < 0.05), whereas the IgG level in the milk increased in linear and quadratic manners (*P* < 0.05) as dietary α-GML content increased (Table 4). No differences (*P* > 0.05) in concentrations of IgM in colostrum and milk were detected among the four groups of sows.

**Table 4:** Effects of α-glycerol monolaurate (α-GML) supplementation during late gestation and lactation on immunoglobulin levels in colostrum and milk of sows

Item	Dietary α-GML level, mg/kg				SEM	*P*-value		
	0	500	1000	2000		Treatment	Linear	Quadratic
Colostrum								
IgA, mg/mL	11.61	13.04	13.78	11.25	0.30	< 0.05	0.46	< 0.05
IgG, mg/mL	66.76	71.18	71.28	70.42	0.67	< 0.05	0.13	< 0.05
IgM, mg/mL	5.22	5.19	5.37	5.39	0.09	0.85	0.46	0.94
Milk								
IgA, mg/mL	3.02	3.29	3.38	2.95	0.09	0.30	0.29	< 0.05
IgG, mg/mL	27.69	31.41	33.27	30.97	0.76	0.06	< 0.05	< 0.05
IgM, mg/mL	5.34	5.25	5.23	5.31	0.06	0.87	0.92	0.42

Values are means with SEM, *n* = 6 sows/treatment group

### Fatty acid profiles of sows in colostrum and milk

Fatty acid profiles in colostrum and milk of sows were summarized in Tables 5 and 6. In colostrum, C12:0, C15:0, C17:0, C18:2n6c, C18:3n3, C24:0, and C22:6n3 increased (*P* < 0.05) in linear and quadratic manners, C20:3n6 increased in quadratic manner and C20:5n3 increased in linear manner (*P* < 0.05), C8:0 decreased (*P* < 0.05) in linear manner as dietary α-GML content increased. In milk, C12:0, C18:3n3, and C20:5n3 increased (*P* < 0.05) in linear and quadratic manners, C22:0 increased (*P* < 0.05) in linear manner, and C23:0, C24:0, and C24:1 increased (*P* < 0.05) in quadratic manner as dietary α-GML content increased. Moreover, C8:0, C10:0, and C14:0 decreased (*P* < 0.05) in linear and quadratic manners, and C14:1 decreased (*P* < 0.05) in quadratic manner, and C20:3n6 decreased (*P* < 0.05) in linear manner in dietary α-GML supplemented sow milk compared with the control sows.

**Table 5:** Effects of α-glycerol monolaurate (α-GML) supplementation during late gestation and lactation on fatty acid profiles in colostrum of sows

Item	Dietary α-GML level, mg/kg				SEM	*P*-value		
	0	500	1000	2000		Treatment	Linear	Quadratic
Higher								
C12:0	0.04	0.08	0.12	0.23	0.02	< 0.05	< 0.05	< 0.05
C15:0	0.15	0.15	0.16	0.19	0.01	< 0.05	< 0.05	< 0.05
C17:0	0.24	0.25	0.25	0.28	0.01	0.07	< 0.05	< 0.05
C18:2n6c	29.69	28.44	28.67	31.89	0.50	< 0.05	< 0.05	< 0.05
C18:3n3	2.07	2.25	2.18	2.49	0.05	< 0.05	< 0.05	< 0.05
C20:3n6	0.61	0.68	0.70	0.64	0.01	< 0.05	0.53	< 0.05
C20:5n3	0.23	0.22	0.25	0.26	0.01	0.09	< 0.05	0.11
C24:0	0.17	0.16	0.17	0.21	0.01	< 0.05	< 0.05	< 0.05
C22:6n3	0.61	0.59	0.62	0.70	0.02	0.08	< 0.05	< 0.05
Lower								
C8:0	0.12	0.14	0.09	0.05	0.02	0.11	< 0.05	0.08
Unchanged								
C6:0	0.02	0.02	0.02	0.02	0.00	0.50	0.86	0.96
C10:0	0.01	0.01	0.01	0.01	0.00	0.95	0.92	0.88
C14:0	1.82	1.68	1.56	1.72	0.04	0.23	0.51	0.11
C14:1	0.03	0.03	0.02	0.03	0.00	0.53	0.50	0.46
C16:0	22.24	21.34	21.57	21.38	0.18	0.26	0.18	0.24
C16:1	3.10	2.77	2.67	2.79	0.08	0.24	0.25	0.12
C18:0	4.65	4.48	4.76	4.48	0.06	0.20	0.50	0.61
C18:1n9c	32.02	33.29	32.30	31.23	0.42	0.41	0.30	0.34
C20:0	0.15	0.16	0.15	0.16	0.00	0.36	0.34	0.54
C20:1	0.26	0.37	0.37	0.32	0.02	0.12	0.50	0.07
C21:0	0.33	0.28	0.32	0.31	0.01	0.45	0.88	0.79
C20:4n6	1.15	1.00	0.97	1.02	0.04	0.35	0.34	0.20
C20:3n3	0.15	0.16	0.16	0.17	0.00	0.13	0.09	0.08
C22:0	0.12	0.12	0.12	0.13	0.00	0.66	0.63	0.44
C22:1n9	0.25	0.25	0.23	0.27	0.01	0.27	0.23	0.22
C22:2	0.03	0.03	0.03	0.03	0.00	0.08	0.08	0.21
C23:0	0.07	0.07	0.07	0.07	0.00	0.51	0.19	0.32
C24:1	0.26	0.25	0.26	0.26	0.01	0.86	0.58	0.81
n-3 PUFA	3.06	3.23	3.21	3.61	0.06	< 0.05	< 0.05	< 0.05
n-6 PUFA	31.20	29.76	29.98	33.25	0.50	< 0.05	0.06	< 0.05
n-6:n-3 PUFA ratio	10.29	9.24	9.40	9.25	0.23	0.31	0.19	0.24

Values are means with SEM, *n* = 6 sows/treatment group, *PUFA* Polyunsaturated fatty acid

**Table 6:** Effects of α-glycerol monolaurate (α-GML) supplementation during late gestation and lactation on fatty acid profiles in milk of sows

Item	Dietary α-GML level, mg/kg				SEM	*P-*value		
	0	500	1000	2000		Treatment	Linear	Quadratic
Higher								
C12:0	0.36	0.46	0.61	0.77	0.04	< 0.05	< 0.05	< 0.05
C18:3n3	1.40	1.54	1.61	1.58	0.03	< 0.05	< 0.05	< 0.05
C20:5n3	0.19	0.21	0.23	0.24	0.01	< 0.05	< 0.05	< 0.05
C22:0	0.05	0.06	0.06	0.04	0.00	0.10	0.46	< 0.05
C23:0	0.02	0.02	0.02	0.02	0.00	0.15	< 0.05	0.06
C24:0	0.09	0.10	0.12	0.12	0.01	0.18	< 0.05	0.09
C24:1	0.08	0.09	0.10	0.10	0.00	0.13	< 0.05	0.06
Lower								
C8:0	0.05	0.04	0.04	0.04	0.00	0.06	< 0.05	< 0.05
C10:0	0.32	0.12	0.23	0.20	0.01	< 0.05	< 0.05	< 0.05
C14:0	4.33	3.71	3.71	3.79	0.04	< 0.05	< 0.05	< 0.05
C14:1	4.33	3.71	3.71	3.79	0.04	< 0.01	< 0.05	0.09
C20:3n6	0.09	0.08	0.07	0.09	0.00	< 0.05	0.68	< 0.05
Unchanged								
C6:0	0.05	0.05	0.06	0.05	0.00	< 0.05	0.65	0.14
C15:0	0.11	0.09	0.11	0.10	0.00	0.09	0.85	0.71
C16:0	34.65	32.29	33.89	32.08	0.38	< 0.05	0.06	0.17
C16:1	12.69	10.23	12.84	11.74	0.46	< 0.05	0.90	0.89
C17:0	0.13	0.16	0.14	0.15	0.01	0.33	0.49	0.56
C18:0	3.69	3.84	3.48	3.60	0.05	0.06	0.23	0.43
C18:1n9c	22.76	26.14	22.56	25.34	0.71	0.18	0.45	0.76
C18:2n6c	19.20	17.88	18.01	18.34	0.22	0.12	0.34	0.08
C20:0	0.12	0.12	0.12	0.13	0.00	0.48	0.39	0.64
C20:1	0.21	0.21	0.18	0.20	0.01	0.77	0.65	0.73
C21:0	0.30	0.25	0.25	0.28	0.01	0.28	0.67	0.18
C20:4n6	0.35	0.39	0.33	0.40	0.01	0.23	0.36	0.50
C20:3n3	0.06	0.06	0.05	0.07	0.00	0.15	0.23	0.23
C22:1n9	0.05	0.07	0.05	0.06	0.00	0.08	0.35	0.64
C22:2	0.04	0.04	0.04	0.05	0.00	0.24	0.13	0.26
C22:6n3	0.23	0.30	0.25	0.28	0.01	< 0.05	< 0.05	< 0.05
n-3 PUFA	1.88	2.11	2.13	2.18	0.30	< 0.05	< 0.05	< 0.05
n-6 PUFA	19.68	18.38	18.45	18.88	0.22	0.12	0.38	0.08
n-6:n-3 PUFA ratio	10.51	8.72	8.66	8.68	0.22	< 0.05	< 0.05	< 0.05

Values are means with SEM, *n* = 6 sows/treatment group, *PUFA* Polyunsaturated fatty acid

### Serum immunoglobulin levels of piglets

Effects of α-GML supplementation on levels of IgA, IgG, and IgM in the serum of piglets are summarized in Table 7. Concentrations of IgG and IgM in serum from piglets tended to increase (*P* < 0.10) in quadratic manner, whereas the concentration of IgA tended to increase (*P* < 0.10) in linear manner.

**Table 7:** Effects of α-glycerol monolaurate (α-GML) supplementation during late gestation and lactation on serum immunoglobulin levels on d 21 of piglets

Item	Dietary α-GML level, mg/kg				SEM	*P*-value		
	0	500	1000	2000		Treatment	Linear	Quadratic
IgA, μg/mL	10.53	10.88	11.01	10.63	0.22	0.87	0.07	0.17
IgG, mg/mL	5.03	5.55	5.48	5.19	0.11	0.29	0.59	0.07
IgM, μg/mL	5.04	5.23	6.70	5.43	0.248	0.07	0.73	0.08

Values are means with SEM, *n* = 6 piglets/treatment group

### Fecal microbiota community of piglets

**Fig. 1:**
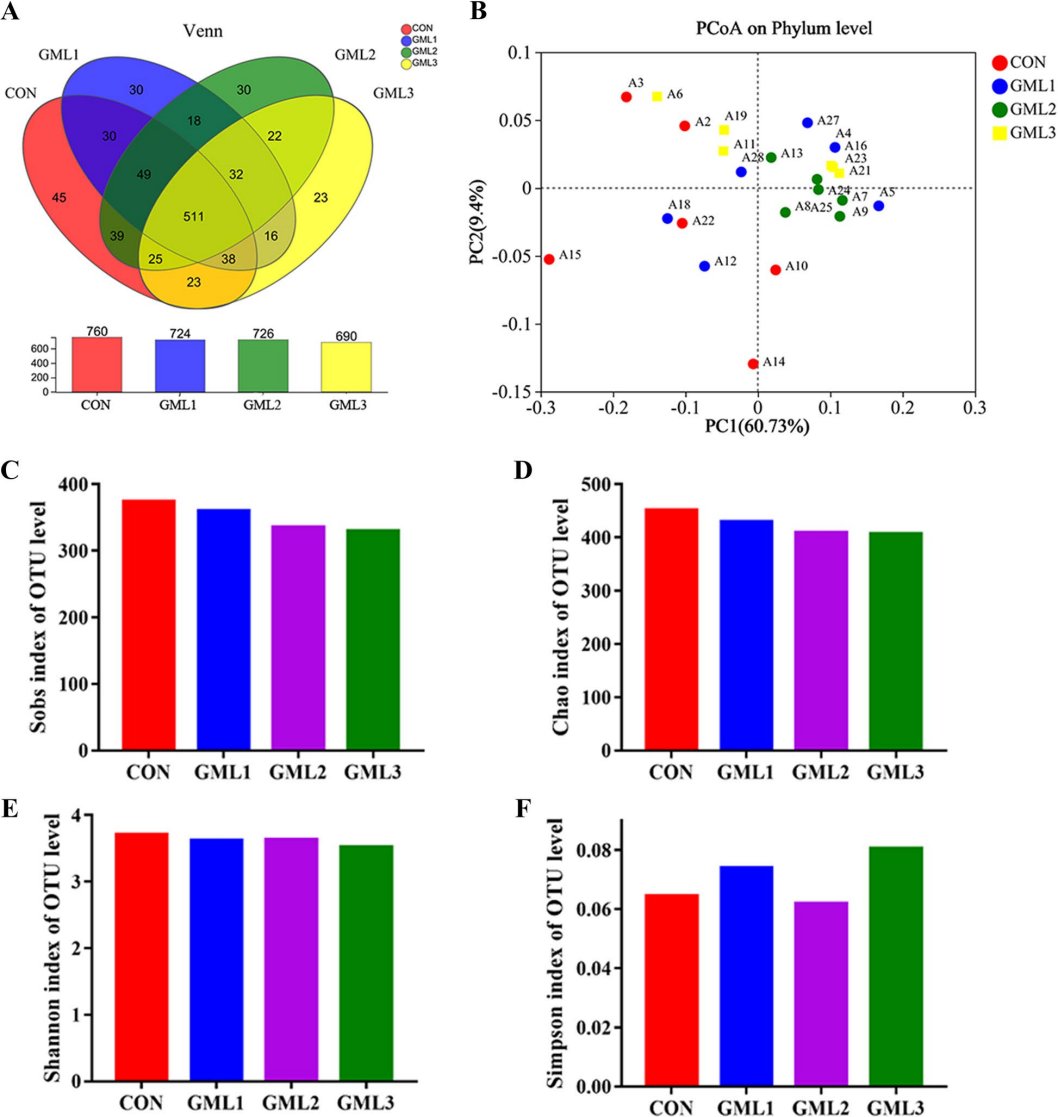
Fecal microbial composition and diversity of suckling piglets. **A** The bacterial operational taxonomic units (OUTs) community composition of the feces in suckling piglets; **B** β-diversity analysis was performed using the unweighted version of the UniFrac-based PcoA; **C**–**F** α-diversity analysis based on indices of Sobs, Chao, Shannon, and Simpson. Data were means ± SD (*n* = 6); CON, control group; GML1, 500 mg/kg α-GML supplemental group; GML2, 1000 mg/kg α-GML supplemental group; GML3, 2000 mg/kg α-GML supplemental group

**Fig. 2:**
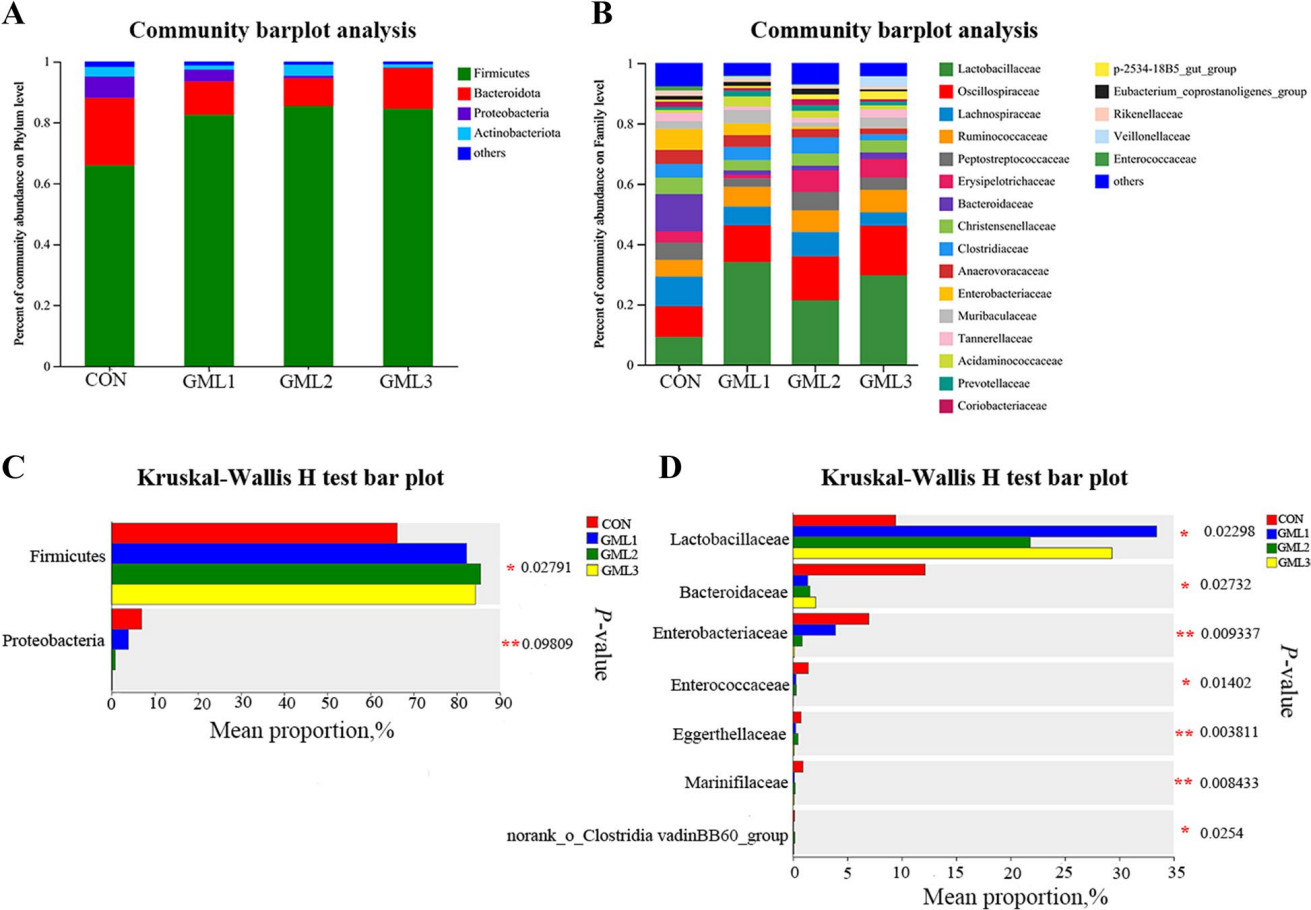
Differences in fecal microbial phylum level and genus level of suckling piglets. **A** and **C** Fecal microbial composition of suckling piglets at phylum level; **B** and **D** Fecal microorganisms of suckling piglets differ at the family level. Data were means ± SD (*n* = 6); ^*^P < 0.05 and ^**^P < 0.01 compared with the CON group. CON, control group; GML1, 500 mg/kg α-GML supplemental group; GML2, 1000 mg/kg α-GML supplemental group; GML3, 2000 mg/kg α-GML supplemental group

There were 760, 724, 726, and 690 OTUs obtained from piglets in 0, 500, 1000, and 2000 mg/kg α-GML groups, respectively, of which 511 were common OTUs (Fig. 1A). Besides, a total of 128 unique OTUs were detected within the four groups. Principal coordinate analysis (PCoA) showed significant clustering characteristics of fecal microbial composition between control and α-GML groups (Fig. 1B). The α-diversity indexes including Sobs, Chao, Shannon, and Simpson indexes were not affected by dietary treatments (Fig. 1C–F). At the phylum level, Firmicutes and Bacteroides were the dominant phyla in the fecal microbial composition of suckling piglets representing approximately 90%, followed by Proteobacteria and Actinobacteriota (Fig. 2A). The relative abundance of Firmicutes was increased (*P* < 0.05), while the relative abundance of Proteobacteria was decreased (*P* < 0.05) in piglets from α-GML-fed sows (Fig. 2B). At the family level, Lactobacillaceae and Oscillospiraceae were the dominant families, followed by Lachnospiraceae, Ruminococcaceae, Peptostreptococcaceae, Erysipelotrichaceae, Bacteroidaceae, Christensenellaceae, Clostridiaceae, etc. (Fig. 2C). The relative abundance of Lactobacillaceae was increased (*P* < 0.05), while the relative abundance of Bacteroidaceae, Enterobacteriaceae, Enterococcaceae, Eggerthellaceae, Marinifilaceae, and norank_o__Clostridia_vadinBB60_group were decreased (*P* < 0.05) in piglets from α-GML-fed sows (Fig. 2D).

## Discussion

Sows are mobilizing body fat and protein to provide precursors and energy for milk production when nutrient intake fails to meet energy requirements [24]. This results in a negative effect on the litter growth and subsequent reproductive performance [25]. Gatlin et al. [26] observed that supplementing with 10% MCT for lactating sows decreased feed intake, in comparison with the control group. The explanation may be that lipid supplementation improved the caloric intake of lactating sows, and the voluntary feed intake of lactation sows was affected by the energy density of the diet [27, 28]. In the present study, maternal α-GML supplementation improved the growth performance of piglets compared with the control group, which was consistent with Gatlin et al. [26], who observed that the growth performance was increased in piglets from MCT-fed sows. It is supposed that this improvement was related to the antibacterial effects, potent anti-inflammatory, or energy density of the diets [14, 29].

In the present study, greater IgA and IgG levels in colostrum and milk of sows-fed α-GML diets, which was consistent with Chen et al. [6], who observed that supplementing the sows’ diet with 7.75 g coated MCFA/kg increased the contents of IgA, IgG, and IgM in colostrum. The increased immunoglobulin in colostrum and milk could enhance the immune status of suckling piglets, which may further improve the growth performance. Previous study has shown that GML could alter the lipid dynamics of human T cells, leading to a reduction in cytokine production, such as IL-2, IFN-γ, and TNF-α [30]. Therefore, the increased IgA and IgG levels may be related to the change in the inflammatory environment induced by α-GML.

The present experiment confirmed that the fatty acid profile in sow’s colostrum was influenced by the dietary α-GML. Previous studies have shown that the impact of dietary fatty acid composition during the last gestation and lactation on fatty acid profile of colostrum and milk [7, 8]. However, this study is the first one using α-GML for sow nutrition. We found that dietary α-GML inhibited de novo lipogenesis of the mammary gland (lower percentage of the sum of C8:0, C10:0, C12:0, and C14:0 in milk) [31]. Consistent with the experimental diet treatment, C12:0 in colostrum and milk was higher from sows-fed α-GML than the control sows. As the sole food source, α-GML from milk for piglets can improve growth performance by improving intestinal morphology and immune status and ameliorating gut microbiota, this was confirmed by our previous study [15]. The odd-chain fatty acids (C15:0, C17:0) mainly exist in milk of ruminants and are mostly synthesized by bacteria [32]. Goat mammary gland can also synthesize these odd-chain fatty acids [33]. Few studies have reported the presence of odd-chain fatty acids in sow milk. In this study, we found that in sow colostrum, C15:0 and C17:0 take about 0.39% of all fatty acids, and α-GML improved their proportion to 0.40%–0.47% in colostrum. In contrast to ruminants, these precursor volatile fatty acids (VFA) are not present in sufficient amounts to produce milk fatty acids. Mo et al. [34] showed that GML could enhance the content of fecal short-chain fatty acids (SCFAs) by anticolitis effect. Propionate can enhance odd-chain fatty acids production, because they can be used to generate propionyl-CoA, which is the precursor for the biosynthesis of odd-chain fatty acids [35]. Thus, it cannot be excluded that a shift towards propionate production, under a different balance of gut microbiota, due to α-GML addition could contribute to these variations in odd number fatty acids. The fatty acid composition of the milk reflects the nutritional status of sows during gestation and lactation as previously described [36]. The inclusion of α-GML in the sow diets was reflected in the higher proportions of C18:2n6c, C18:3n3, C20:3n6, C20:5n3, and C22:6n3 in colostrum. Moreover, C18:3n3 and C20:5n3 were higher in milk in sows-fed α-GML diets than in the control sows. Given that long-chain fatty acids cannot be synthesized in the body, they are mainly supplied by soy oil in the diet, we speculated that α-GML supplementation altered the composition of long-chain fatty acids in milk by improving the ATTD of CF of sows. Moreover, the presence of higher levels of these fatty acids elevated the proportion of n-3 PUFA and n-6 PUFA in colostrum and n-3 PUFA in milk. Higher ratio of n-6:n-3 may limit the production of anti-inflammatory eicosanoids derived from eicosapentaenoic acid [37]. Maternal diets with varying n-6:n-3 PUFA ratios affected the antioxidant status, immune cell eicosanoid responses, immunoglobulins, fatty acids composition, and growth performance [38, 39]. Previous studies have shown the lower ratio of n-6:n-3 PUFA could improve the growth performance in weaned piglets [40] and reproductive function in boar [41].

The gut microbiota in the gastrointestinal tract has numerous roles benefiting the host, such as the production of vitamins, maintenance of normal functions of the intestinal villi, and regulation of the immune responses [42]. Firmicutes and Bacteroidota play key roles in creating and maintaining an anaerobic environment in the intestinal tract of piglets with a relative abundance approximately of 90% in this study, which was consistent with the previous report on suckling piglets [43]. *Desulfovibrio* in Proteobacteria has been shown to further induce increased intestinal permeability and destruction of intestinal barrier structure by reducing sulfate to sulfide and inhibiting the growth of intestinal epithelial cells [44, 45]. In the present study, maternal α-GML supplementation increased the abundance of Firmicutes and decreased the abundance of Proteobacteria in the feces of suckling piglets, which indicated that α-GML can modulate the structure and diversity of intestinal microbiota in suckling piglets. The Lactobacillaceae family, belonging to the Firmicutes phylum, is considered a beneficial microbe to improve growth performance by modulating gut health and immunity [46]. Our results showed that Lactobacillaceae was increased in piglets from α-GML-fed sows, which could explain the improvement of growth and immunity. Enterobacteriaceae and Enterococcus, as aerobic or facultative anaerobic bacteria, mainly settle in the intestinal tract of piglets on d 1 and 3 [47]. With the further development of the piglet intestinal flora, the anaerobic environment formed in the piglet intestinal tract is favorable for obligate anaerobic bacteria such as *Bacteroides*, *Lachnospira*, and *Clostridium*, and the formation of a bacterial community structure with Firmicutes and Bacteroidetes as dominant bacteria accompanied by a decrease in the abundance of Proteobacteria [47]. In this study, a higher abundance of Enterobacteriaceae and Enterococcaceae in the feces of piglets in the control group that only appeared in the intestinal tract of piglets in the early stage of birth, which to some extent indicated that the intestinal development of piglets was not perfect, and there was a higher risk of opportunistic pathogens infection. Together, these findings indicated that the maternal α-GML could influence the composition of the intestinal microbiota in suckling piglets.

## Conclusions

In conclusion, α-GML supplementation could altere immunoglobulins and fatty acid composition in milk, thus ameliorating intestinal microbe in suckling piglets, which may contribute to improving the growth performance in suckling piglets.

## Abbreviations

α-GML: Alpha-glycerol monolaurate
ADFI: Average daily feed intake
ASFv: African swine fever virus
FDA: Food and Drug Administration
GRAS: Generally Recognized as Safe
IgA: Immunoglobulin A
IgG: Immunoglobulin G
IgM: Immunoglobulin M
LDA: Linear discriminant analysis
MCFA: Medium-chain fatty acid
MCT: Medium-chain triglyceride
OTUs: Operational taxonomic units
PCoA: Principal coordinate analysis
PUFA: Polyunsaturated fatty acid
SCFAs: Short-chain fatty acids
VFA: Volatile fatty acid
